# A Microwave-Assisted Bismuth Nitrate-Catalyzed Unique Route Toward 1,4-Dihydropyridines

**DOI:** 10.3390/molecules17032643

**Published:** 2012-03-05

**Authors:** Debasish Bandyopadhyay, Stephanie Maldonado, Bimal K. Banik

**Affiliations:** Department of Chemistry, The University of Texas-Pan American, 1201 West University Drive, Edinburg, TX 78539, USA

**Keywords:** microwave, 1,4-dihydropyridines, catalysis, green synthesis, bismuth nitrate

## Abstract

The classical Hantzsch reaction is one of the simplest and most economical methods for the synthesis of biologically important and pharmacologically useful 1,4-dihydropyridine derivatives. Bismuth nitrate pentahydrate under microwave irradiation is proven to act as a very efficient catalyst for a one-pot, three-component synthesis of 1,4-dihydropyridines in excellent yields from diverse amines/ammonium acetate, aldehydes and 1,3-dicarbonyl compounds within 1–3 min under solvent-free conditions. The present environmentally benign procedure for the synthesis of 1,4-dihydropyridines is suitable for library synthesis and it will find application in the synthesis of potent biologically active molecules. The excellent yield and extreme rapidity of the method is due to a concurrent effect of the catalyst and microwave irradiation.

## 1. Introduction

In multicomponent reactions (MCRs), more than two reactants combine in a sequential manner to produce in a highly selective fashion a product that retains the majority of the atoms of the starting materials. In a MCR, a product is assembled according to a cascade of elementary chemical reactions. Thus, there is a network of reaction equilibria, which all finally flow into an irreversible step yielding the final product. Such processes are of great interest in diversity-oriented synthesis, especially to generate compounds libraries for screening purposes [1,2,3,4,5,6]. The Hantzsch reaction [7], the oldest known synthesis of 1,4-dihydropyridines (1,4-DHPs) and their derivatives, is one of the most general methods (changed) useful for the synthesis of a number of medicinally and pharmacologically molecules which includes commercial drugs such as nifedipine [8], felodipine [9], nicardipine [10] and amlodipine [11] which are used in treatment of angina and hypertension. It has been reported [12,13,14] that the pharmaceutical action of these drugs is related to binding to voltage dependent L-type of calcium channel and thus decreasing the passage of Ca^2+^ ions to the cell. The result is relaxation of smooth muscle cells and lowering of the blood pressure. An alternate mechanism on a molecular level has been found and is based on increased NO release from the intact endothelium [15]. In addition, 1,4-dihydropydines have been reported as anticancer [16], neurotropic [17], glycoprotein inhibitors [18], bronchodilating [19], and antidiabetic [20] agents.

Recently a number of publications have appeared on the synthesis of 1,4-dihydropyridines [21,22,23,24,25,26,27]. Nonetheless, this reaction is still under active investigation because of the importance of the dihydropyridine products. A number of new catalysts, solid supports and solvents have been used for this reaction. Some recent examples include molecular iodine [28], ruthenium trichloride [29], lithium bromide [30], Zn[(L)proline]_2_ [31], nano aluminium nitride [32], bakers’ yeast [33], Wells-Dawson heteropolyacid (H_6_P_2_W_18_O_62_·24H_2_O) [34], silica gel supported sodium bisulfate [35], solvents like trifluoroethanol [36], and ionic liquid [37]. Many of these reported methods involve the use of expensive reagents, hazardous solvents, long reaction times and tedious workup procedures. Therefore, it is desirable to develop a rapid, efficient and practical method for the synthesis of 1,4-dihydropyridines under eco-friendly conditions.

**Scheme 1:**
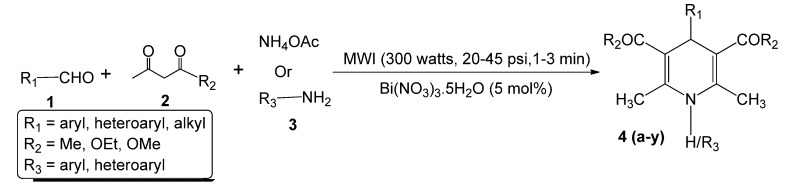
Microwave-assisted one-pot, three component synthesis of 1,4-dihydropyridines using bismuth nitrate as catalyst (5 mol%) under solventless condition.

We report herein an easy and extremely rapid method for the preparation 1,4-dihydropyridines under solvent-free conditions in the presence of catalytic amounts of bismuth nitrate under microwave irradiation. In contrast to the existing methods, our method is extremely rapid, simple and high yielding (Scheme 1).

## 2. Results and Discussion

### 2.1. Results

We have been engaged in the study of microwave-induced reactions for many years. Using microwave irradiation technique we have successfully developed several new methodologies which include stereoselective synthesis of β-lactams [38,39,40], synthesis of pyrroles [41,42,43,44], aza-Michael addition [45], synthesis of quinoxalines [46], *etc.* On the other hand, organo-bismuth chemistry [47,48,49,50] is considered an emerging field of research in synthetic organic chemistry. We have demonstrated the catalytic activity of trivalent bismuth nitrate pentahydrate in a number of examples. These experiments resulted in various methods that include nitration of aromatic systems [51,52,53], Michael reactions [54], protection of carbonyl compounds [55], deprotection of oximes and hydrazones [56], Paal-Knorr synthesis of pyrroles [57], hydrolysis of amides [58], electrophilic substitution of indoles [59,60], synthesis of α-aminophosphonates [61], and Biginelli condensation [62]. During the course of present study, it has been conceived that 1,4-dihydropyridine derivatives can be easily prepared using bismuth nitrate as the catalyst. Our success in the bismuth nitrate-induced reaction has revealed that this reagent acts as a Lewis acid. Moreover, it has been discovered that this reagent is compatible with the presence of sensitive functional groups. This idea has been extended in this paper to the reaction on carbonyl compounds (both aldehydes and 1,3-dicarbomyl compounds) with a suitable ammonia source in the presence of catalytic amounts of bismuth nitrate under solvent-free conditions. 

Our initial work started with screening of catalyst loading and solvent so as to identify optimal reaction conditions for the synthesis of 1,4-dihydropyridines. First of all, a number of bismuth salts, e.g., bismuth chloride, bismuth triflate, bismuth subnitrate, bismuth bromide, bismuth iodide and bismuth nitrate pentahydrate have been screened using benzaldehyde, ethyl acetoacetate and ammonium acetate as a model reaction under automated CEM microwave irradiation conditions (300 Watts, 50 °C, 1 min). The results are shown in Table 1.

**Table 1:** Microwave-assisted one-pot, three component synthesis of 1,4-dihydropyridines from benzaldehyde (1 mmol), ethyl acetoacetate (2 mmol) and ammonium acetate (1 mmol) using bismuth-salts as catalyst (10 mol%) under solventless condition (1 min): catalyst optimization.

Entry	Bi-salt (10 mol%)	Yield (%) ^a^
1	BiCl_3_	81
2	Bi(OTf)_3_	79
3	BiI_3_	72
4	Bi_5_O(OH)_9_(NO_3_)_3_	67
5	BiBr_3_	73
6	Bi(NO_3_)_3_.5H_2_O	96
7	No catalyst	52

^a^ isolated yield.

Bismuth nitrate pentahydrate was found to be the best catalyst under these conditions (Entry 6, Table 1). The reaction proceded in the absence of any catalyst (only microwave irradiation) in 52% yield within a minute (Entry 7, Table 1). When continued for 10 minutes the yield of the desired product could be increased to 64%. The same reaction was used to optimize the amount of the catalyst (Table 2). The results show that 5 mol% bismuth nitrate pentahydrate is required to complete the reaction within one minute (Entry 6, Table 2). The reaction was then performed in various solvents as well as under neat conditions under identical microwave power using bismuth nitrate pentahydrate (5 mol%) as the catalyst to identify the best conditions (Table 3). The results suggest that bismuth nitrate pentahydrate is the best catalyst under neat conditions for the reaction (Entry 8, Table 3). Considering the above observations we next carried out a series of reactions using various aldehydes, carbonyl compounds and aromatic amines/ammonium acetate in presence of bismuth nitrate pentahydrate (5 mol%) under microwave irradiation.

**Table 2:** Microwave-assisted one-pot, three component synthesis of 1,4-dihydropyridines from benzaldehyde (1 mmol), ethyl acetoacetate (2 mmol) and ammonium acetate (1 mmol) using bismuth nitrate pentahydrate as catalyst under solventless condition (1 minute): optimization of the amount of the catalyst.

Entry	Bi (NO_3_)_3_.5H_2_O (mol%)	Yield (%) ^a^
1	30	88
2	25	87
3	20	93
4	15	91
5	10	96
6	5	99
7	2	86
8	1	73

^a^ isolated yield.

**Table 3:** Microwave-assisted one-pot, three component synthesis of 1,4-dihydropyridines from benzaldehyde (1 mmol), ethyl acetoacetate (2 mmol) and ammonium acetate (1 mmol) using bismuth nitrate pentahydrate as catalyst (5 mol%) for 1 minute: Solvent optimization.

Entry	Solvent (1 mL)	Yield (%) ^a^
1	Water	91
2	THF	77
3	Ethanol	79
4	Toluene	62
5	Methanol	76
6	Dichloromethane	71
7	DMSO	76
8	Neat	99

^a^ isolated yield.

Bismuth nitrate pentahydrate, a commercially available solid salt, is very economical and much less toxic than other Lewis acids. It is very convenient to conduct reactions with bismuth nitrate because of its stability in the presence of moisture and oxygen. In all the cases, the reactions were completed within 1–3 min and the products were obtained in excellent yield (Table 4).

### 2.2. Discussion

A series of 1,4-dihydropyridines were synthesized by using diverse aldehydes, 1,3-diketo compounds and ammonium acetate/amines in the presence of bismuth nitrate pentahydrate (5 mol%) as catalyst under microwave irradiation. As shown in Table 4, the reaction proceeded equally well irrespective of the nature of the carbonyl compounds (aliphatic, aromatic, heteroaromatic) or amines (aromatic, heteroaromatic) to afford the corresponding products in excellent yield (87%–99%). The catalytic system worked well with acid-sensitive heteroaromatic aldehyde (Entries 17-22, Table 4), α, β-unsaturated aldehyde (Entry 23, Table 4) and aliphatic aldehydes (Entries 24–25, Table 4). Aromatic primary amines (Entries 4, 6, 8, 9, 14, 20 and 22, Table 4) and heterocyclic amines (Entries 5 and 21, Table 4) upon reaction with aldehydes and 1,3-diketo compounds produced the corresponding products in excellent yields. When ammonium acetate was used as ammonia source (Entries 1–3, 7, 10–13, 15–19, 23–25, Table 4) equally excellent yields of the corresponding products were isolated. Importantly, all reactions were completed within 1–3 min (Table 4). Tajbakhsh *et al.* reported [63] a bismuth nitrate-induced oxidation of Hantzsch 1,4-dihydropyridines in presence of solid support (silica gel) under microwave (kitchen) irradiation. However, such oxidation was completely avoided using controlled microwave irradiation (300 Watts, 50 °C, 24–45 psi) under neat conditions (no solid support or solvent).

**Table 4:** Microwave-assisted one-pot, three component synthesis of 1,4-dihydropyridines using bismuth nitrate as catalyst (5 mol%) under solventless condition following Scheme 1.

Entry	Aldehyde	1,3-Dicarbonyl	Ammonia	Product	Time	Yield (%) ^a^
		compounds	source		(min)	
**1**	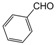	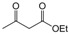	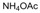	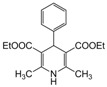	**1**	**99**
**2**	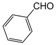	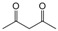	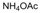	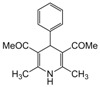	**2.5**	**91**
**3**	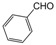	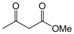	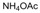	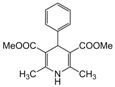	**2**	**93**
**4**	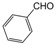	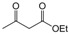	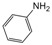	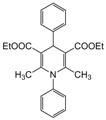	**1.5**	**94**
**5**	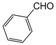	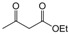	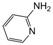	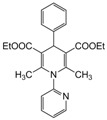	**2.5**	**90**
**6**	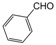	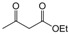	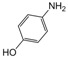	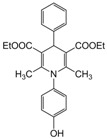	**3**	**91**
**7**	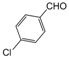	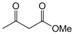	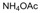	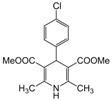	**1**1.5	**99**
**8**	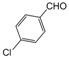	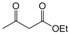	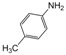	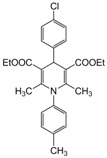	**2**	**89**
**9**	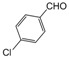	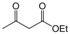	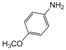	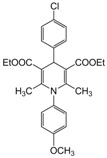	**2.5**	**94**
**10**	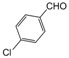	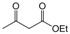	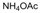	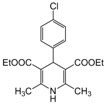	**2**	**97**
**11**	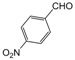	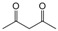	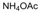	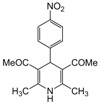	**3**	**84**
**12**	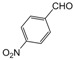	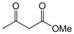	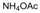	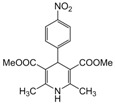	**3**	**91**
**13**	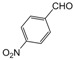	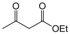	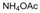	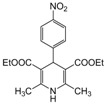	**3**	**93**
**14**	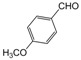	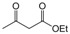	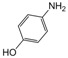	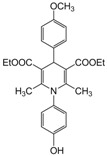	**3**	**89**
**15**	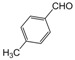	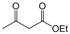	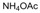	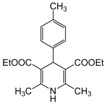	**2.5**	**98**
**16**	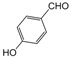	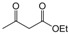	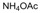	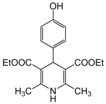	**3**	**95**
**17**	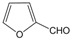	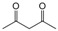	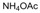	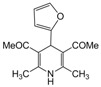	**2**	**95**
**18**	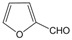	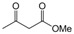	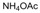	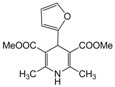	**2**	**93**
**19**	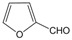	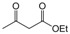	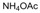	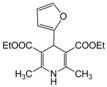	**2**	**98**
**20**	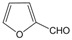	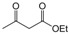	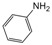	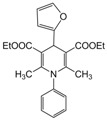	**2.5**	**97**
**21**	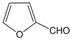	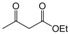	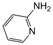	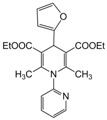	**3**	**90**
**22**	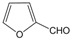	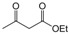	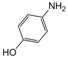	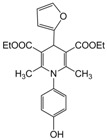	**2.5**	**92**
**23**	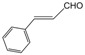	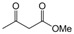	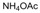	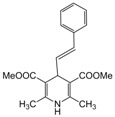	**3**	**91**
**24**	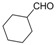	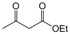	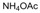	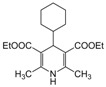	**3**	**86**
**25**	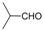	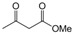	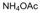	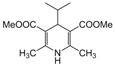	**3**	**87**

^a^ isolated yield.

The selectivity observed in our current method for the preparation of 1,4-dihydropyridines is interesting because further oxidation to pyridine derivatives could be prevented under control of the exposure to microwave irradiation and judicial choice of the catalyst. Although we have had success in the preparation of 1,4-dihydropyridines following the above method, however, the mechanism has yet to be established. We have been working on the synthesis and biological evaluation of various β-lactams as novel anticancer agents [64,65,66,67,68,69,70] over the past several years. The synthesis of β-lactams through imines requires a carbonyl compound and an amine. Our study in this field, suggests that carbonyl compounds, amines/ammonium acetate and 1,3-dicarbonyl compounds in the presence of Lewis acid will produce 1,4-dihydropyridines in excellent yield. This hypothesis has been tested by reacting several carbonyl compounds and 1,3-dicarbonyl compounds using aromatic amine/ammonium acetate as ammonia source in the presence of catalytic amount of bismuth nitrate pentahydrate (Table 4) following Scheme 1. Although the mechanism of the reaction has not been studied in detail, based on the structure of the starting materials and products, a plausible mechanistic pathway may be suggested as follows.

**Scheme 2:**
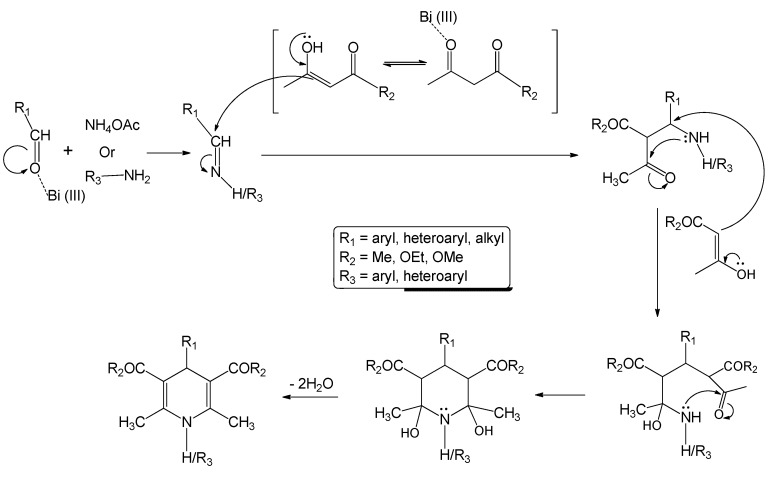
Plausible mechanistic pathway for the synthesis of 1,4-dihydropyridines.

In the initial step, bismuth nitrate acts as Lewis acid to facilitate the formation of the corresponding imine through a condensation of the amine/ammonium acetate and aldehyde. The 1,3-diketo compound is also activated due to the presence of bismuth nitrate and the equilibrium is shifted toward the enol form. As a result, a nucleophilic attack to imine carbon can take place. A second nucleophilic attack by another enol tautomer to the same carbon, subsequent ring-closure and dehydration yield the product (Scheme 2). Microwaves, as a part of electromagnetic spectrum, are composed of two field components: electric and the magnetic. For the purpose of heating, the electric component is important as it results in a force being applied to all the polar or charged species. Such species, in response to the electric field, start to move or rotate and this causes additional polarization of the polar species in the vicinity. When dipolar species are subjected to the electric component of microwave fields they start to oscillate, following the oscillation of the electric field. During such oscillation, the polar or charged species collide with neighboring particles (charged or neutral). This rapid motion and resulting intermolecular friction cause an intense internal heat that can increase the rate of reaction [71]. It is obvious that the dielectric properties of the material under consideration are of paramount importance. In presence of microwaves, bismuth nitrate increases the “anionic activation” [72]. On this basis, the relative permittivity (ability of a molecule to be polarized by the application of an electric field) of the carbonyl groups (aldehyde as well as the diketo compound) (Scheme 2) increases, which facilitates microwave heating extensively. The larger the relative permittivity of a substance, the greater will be the coupling with microwaves [73]. When the reagents (amine, aldehyde and 1,3-diketo compound) and the catalyst (bismuth nitrate pentahydrate) are subjected to microwave irradiation, microwaves passes through the (glass) walls of the reaction vessel and heat only the reactants avoiding local overheating at the reaction walls. This can eliminate reaction side products and helps to explain the higher yields and purities. The extreme rapidity with excellent yield of the reaction can be rationalized as a synergistic effect of the Lewis acid catalyst (bismuth nitrate) and microwave irradiation.

## 3. Experimental

### 3.1. General

Melting points were determined in a Fisher Scientific electrochemical Mel-Temp* manual melting point apparatus (Model 1001) equipped with a 300 °C thermometer. FT-IR spectra were registered on a Bruker IFS 55 Equinox FTIR spectrophotometer as KBr discs. ^1^H-NMR (600 MHz) and ^13^C-NMR (150 MHz) spectra were obtained at room temperature with Bruker superconducting Ultrashield^TM^ Plus 600 MHz NMR spectrometer with central field 14.09 Tesla, coil inductance 89.1 Henry and magnetic energy 1127.2 kJ using CDCl_3_ as solvent. Elemental analyses (C, H, N) were conducted using the Perkin-Elmer 2400 series II elemental analyzer, their results were found to be in good agreement (±0.2%) with the calculated values for C, H, N. Bismuth nitrate pentahydrate (reagent grade) 98% (Cat # 248592-500G, Batch # MKBC6772) purchased from Sigma-Aldrich Corporation was used. All other chemicals were purchased from Sigma-Aldrich Corporation (analytical grade). Throughout the project solvents were purchased from Fisher-Scientific. Deionized water was used for the preparation of all aqueous solutions. 

### 3.2. General Procedure for the One-Pot, Three-Component Synthesis of 1,4-Dihydropyridines

A representative experimental procedure (Entry 1, Table 4) is as follows: bismuth nitrate pentahydrate (5 mol%, 24 mg) was added to a mixture of ammonium acetate (1 mmol, 77 mg), benzaldehyde (1 mmol, 0.1 mL) and ethyl acetoacetate (2 mmol, 0.25 mL) in a microwave vial equipped with a small magnetic stir bar. The resulting mixture was stirred under automated CEM microwave irradiation at 50 °C (300 Watts, 20–45 psi) and the progress of the reaction was monitored by TLC every 30 seconds. After completion of the reaction (Table 4) ethyl acetate (5 mL) was added and the solution was filtered and washed successively with brine (3 mL) and water (3 mL). It was dried over anhydrous sodium sulphate and filtered. A short column of silica gel was used to isolate the product 2,6-dimethyl-4-phenyl-1,4-dihydropyridine-3,5-diethylcarboxylate in 99% overall yield.

*2,6-Dimethyl-4-phenyl-1,4-dihydropyridine-3,5-diethylcarboxylate (**Entry 1, Table 4**)*. White solid; mp 158–159 °C; IR (KBr) 3320, 1698, 1653, 1480, 1215, 790 cm^−1^; ^1^H-NMR (CDCl_3_) δ 1.23 (t, *J* = 7.0, 6H), 2.34 (s, 3H), 2.33 (s, 3H), 4.09 (q, *J* = 7.2 Hz, 4H), 4.99 (s, 1H), 5.96 (br s, 1H, NH), 7.09–7.34 (m, 5H); ^13^C-NMR (CDCl_3_) δ 14.08, 19.43, 39.46, 59.41, 102.89, 127.34, 129.76, 136.32, 145.66, 147.10, 165.09. Anal. Calcd for C_19_H_23_NO_4_: C, 69.28; H, 7.04, N, 4.25. Found: C, 69.05; H, 6.98; N, 4.29.

*2,6-Dimethyl-4-phenyl-1,4-dihydropyridine-3,5-diethanone (**Entry 2, Table 4**)*. Yellowish white solid; mp 134–137 °C; IR (KBr) 3362, 3078, 1721, 612 cm^−1^; ^1^H-NMR (CDCl_3_) δ 2.21 (s, 6H), 2.24 (s, 6H), 4.90 (s, 1H), 6.02 (br s, 1H, NH), 7.22–7.36 (m, 5H); ^13^C-NMR (CDCl_3_) δ 22.06, 29.61, 36.87, 115.67, 123.89, 126.32, 129.55, 142.56, 147.11, 197.01. Anal. Calcd for C_17_H_19_NO_2_: C, 75.81; H, 7.11, N, 5.20. Found: C, 75.71; H, 7.14; N, 5.11. 

*2,6-Dimethyl-4-phenyl-1,4-dihydropyridine-3,5-dimethylcarboxylate (**Entry 3, Table 4**)*. White solid; mp 116–118 °C; IR (KBr) cm^−1^; 3362, 3065, 2983, 1705, 1112; ^1^H-NMR (CDCl_3_) δ 2.27 (s, 6H), 3.74 (s, 6H), 4.84 (s, 1H), 7.23–7.34 (m, 5H); ^13^C-NMR (CDCl_3_) δ 20.09, 41.09, 55.77, 105.84, 128.51, 129.08, 131.92, 143.58, 149.43, 166.59. Anal. Calcd for C_17_H_19_NO_4_: C, 67.76; H, 6.36, N, 4.65. Found: C, 67.65; H, 6.29; N, 4.62.

*2,6-Dimethyl-1,4-diphenyl-1,4-dihydropyridine-3,5-diethylcarboxylate (**Entry 4, Table 4**)*. Brownish white solid; mp 161–162 °C; IR (KBr) 3325, 2987, 1722, 1624, 1092 cm^−1^; ^1^H-NMR (CDCl_3_) δ 1.19 (t, *J* = 7.02, 6H), 2.29 (s, 6H), 4.18 (q, *J* = 6.3 Hz, 4H), 4.99 (s, 1H), 7.30–7.56 (m, 10H); ^13^C-NMR (CDCl_3_) δ 16.02, 19.76, 44.34, 62.87, 105.39, 122.32, 123.45, 126.77, 127.47, 128.43, 130.58, 141.55, 144.39, 153.84, 167.09. Anal. Calcd for C_15_H_27_NO_4_: C, 74.05; H, 6.67, N, 3.45. Found: C, 74.11; H, 6.72; N, 3.41.

*2,6-Dimethyl-4-phenyl-1,4-dihydropyridine-1-(2′-pyridyl)-3,5-diethylcarboxylate (**Entry 5, Table 4**)*. White solid; mp 144–146 °C; IR (KBr) 3110, 2961, 1692, 1633, 1041 cm^−1^; ^1^H-NMR (d_6_-DMSO) δ 1.21 (t, *J* = 7.1 Hz, 6H), 2.24 (s, 6H), 4.21 (q, *J* = 7.2 Hz, 4H), 4.82 (s, 1H), 6.89–8.11 (m, 9H); ^13^C-NMR (d_6_-DMSO) δ 15.01, 19.46, 45.71, 63.82, 104.62, 115.48, 124.42, 124.61, 127.91, 129.59, 139.62, 145.85, 148.29, 150.73, 167.57. Anal. Calcd for C_24_H_26_N_2_O_4_: C, 70.93; H, 6.40, N, 6.89. Found: C, 70.87; H, 6.41; N, 6.90.

*2,6-Dimethyl-1-(4-hydroxyphenyl)-4-phenyl-1,4-dihydropyridine-3,5-diethylcarboxylate (**Entry 6, Table 4**)*. Yellow solid; mp 90–91 °C; IR (KBr) 3433, 3055, 2910, 1712, 1698, 1482, 790 cm^−1^; ^1^H-NMR (CDCl_3_) δ 1.22 (t, *J* = 7.2, 6H), 2.29 (s, 6H), 4.14 (q, 4H), 5.11 (s, 1H), 6.92–7.41 (m, 9H); ^13^C-NMR (CDCl_3_) δ 14.91, 20.54, 42.52, 62.41, 104.11, 117.34, 122.64, 125.29, 127.47, 128.76, 134.32, 145.16, 148.51, 153.12, 167.03. Anal. Calcd for C_25_H_27_NO_5_: C, 71.24; H, 6.46, N, 3.32. Found: C, 70.98; H, 6.37; N, 3.38. 

*2,6-Dimethyl-4-(4-chlorophenyl)-1,4-dihydropyridine-3,5-dimethylcarboxylate (**Entry 7, Table 4**)*. Yellow solid; mp 194–195 °C; IR (KBr) 3325, 1701, 1653, 1371, 1210 cm^−1^; ^1^H-NMR (CDCl_3_) δ 2.32 (s, 6H), 3.69 (s, 6H), 4.93 (s, 1H), 5.69 (br. s, 1H), 7.20–7.27 (m, 4H); ^13^C-NMR (CDCl_3_) δ 19.46, 40.01, 52.00, 103.73, 128.14,129.32, 131.56, 144.65, 146.04, 167.89. Anal. Calcd for C_17_H_18_ClNO_4_: C, 60.81; H, 5.40, N, 4.17. Found: C, 60.65; H, 5.43; N, 4.27. 

*2,6-Dimethyl-1-(4-methylphenyl)-4(4-chlorophenyl)-1,4-dihydropyridine-3,5-diethylcarboxylate (**Entry 8, Table 4**)*. Brown solid; mp 102–104 °C; IR (KBr) 3312, 1705, 1681, 1290, 1140 cm^−1^; ^1^H-NMR (CDCl_3_) δ; 1.22–1.26 (m, 6H), 2.05 (s, 6H), 2.41 (s, 3H), 4.11 (m, 4H), 5.01 (s, 1H), 6.98–7.35 (m, 8H); ^13^C-NMR (CDCl_3_) δ 15.63, 20.92, 22.30, 45.76, 61.51, 105.73, 124.72, 128.64, 128.99, 130.02, 131.84, 131.98, 139.41, 143.39, 155.91, 167.09. Anal. Calcd for C_26_H_28_ClNO_4_: C, 68.79; H, 6.22, N, 3.09. Found: C, 68.71; H, 6.27; N, 3.02.

*2,6-Dimethyl-1-(4-methoxyphenyl)-4-(4-chlorophenyl)-1,4-dihydropyridine-3,5-diethcarboxylate (**Entry 9, Table 4**)*. Brown solid; mp 134–136 °C; IR (KBr) 3310, 1702, 1674, 1543, 1201, 972 cm^−1^; ^1^H NMR (CDCl_3_) δ; 1.23–1.25 (m, 6H), 2.06 (s, 6H), 3.85 (s, 3H), 4.11–4.15 (m, 4H), 5.09 (s, 1H), 6.92–7.31 (m, 8H); ^13^C-NMR (CDCl_3_) δ 15.87, 21.49, 45.75, 55.75, 61.54, 104.48, 118.58, 122.74, 127.99, 130.07, 132.38, 134.29, 143.59, 154.20, 155.25, 167.21. Anal. Calcd for C_26_H_28_ClNO_5_: C, 66.45; H, 6.01, N, 2.98. Found: C, 66.37; H, 5.91; N, 2.93.

*2,6-Dimethyl-4-(4-chlorophenyl)-1,4-dihydropyridine-3,5-diethylcarboxylate (**Entry 10, Table 4**)*. White solid; mp 145–146 °C; IR (KBr) 3357, 1697, 1654, 1490, 1210, 1122, 790, 672 cm^−1^; ^1^H-NMR (CDCl_3_) δ 1.24 (t, *J* = 7.2 Hz, 6H), 2.34 (s, 6H), 4.10 (q, *J* = 7.2 Hz, 4H), 4.96 (s, 1H), 5.89 (br s, 1H), 7.20–7.24 (m, 4H); ^13^C-NMR (CDCl_3_) δ 14.39, 19.61, 39.22, 59.90, 103.79, 127.90, 129.41, 131.73, 144.12, 146.32, 167.53. Anal. Calcd for C_19_H_22_ClNO_4_: C, 62.72; H, 6.09, N, 3.85. Found: C, 62.66; H, 6.00; N, 3.81.

*2,6-Dimethyl-4-(4-nitrophenyl)-1,4-dihydropyridine-3,5-diethanone (**Entry 11, Table 4**)*. Yellow solid; mp 130–32 °C; IR (KBr) 3372, 3066, 2980, 1720, 1492, 790, 712 cm^−1^; ^1^H-NMR (CDCl_3_) δ 2.27 (s, 6H), 2.32 (s, 6H), 4.72 (s, 1H), 5.94 (br s, 1H), 7.53 (d, *J* = 8.6 Hz, 2H), 8.13 (d, *J* = 8.6 Hz, 2H); ^13^C-NMR (CDCl_3_) δ 20.76, 27.18, 34.14, 111.08, 124.53, 127.51, 143.83, 147.27, 151.20, 195.26. Anal. Calcd for C_17_H_18_N_2_O_4_: C, 64.96; H, 5.77, N, 8.91. Found: C, 64.90; H, 5.71; N, 9.01.

*2,6-Dimethyl-4-(4-nitrophenyl)-1,4-dihydropyridine-3,5-dimethylcarboxylate (**Entry 12, Table 4**)*. Yellow solid; mp 152–154 °C; IR (KBr) 3364, 3092, 2981, 1698, 1495, 792 cm^−1^; ^1^H-NMR (CDCl_3_) δ 2.31 (s, 6H), 3.75 (s, 6H), 4.89 (s, 1H), 5.91 (br s, 1H), 7.52 (d, *J* = 8.6 Hz, 2H), 8.11 (d, *J* = 8.6 Hz, 2H); ^13^C-NMR (CDCl_3_) δ 21.28, 45.01, 55.37, 105.41, 124.94, 127.40, 142.48, 148.03, 149.73, 167.09. Anal. Calcd for C_17_H_18_N_2_O_6_: C, 58.96; H, 5.24, N, 8.09. Found: C, 58.87; H, 5.16; N, 8.00. 

*2,6-Dimethyl-4-(4-nitrophenyl)-1,4-dihydropyridine-3,5-diethylcarboxylate (**Entry 13, Table 4**)*. Yellowish brown solid; mp 132–134 °C; IR (KBr) 3316, 1705, 1649, 1521, 1215, 1120, 692 cm^−1^; ^1^H-NMR (CDCl_3_) δ 1.22 (t, *J* = 7.2 Hz, 6H), 2.34 (s, 6H), 4.11 (q, *J* = 7.1 Hz, 4H), 5.10 (s, 1H), 6.15 (br s, 1H), 7.51 (d, *J* = 8.6 Hz, 2H), 8.14 (d, *J* = 8.6 Hz, 2H); ^13^C-NMR (CDCl_3_) δ 14.19, 19.53, 40.11, 60.34, 103.04, 123.63, 128.89, 145.04, 146.23, 155.21, 167.18. Anal. Calcd for C_19_H_22_N_2_O_6_: C, 60.95; H, 5.92, N, 7.48. Found: C, 60.87; H, 5.90; N, 7.41.

*2,6-Dimethyl-1-(4-hydroxyphenyl)-4-(4-methoxyphenyl)-1,4-dihydropyridine-3,5-diethylcarboxylate(**Entry 14, Table 4**)*. Brown solid; mp 89–92 °C; IR (KBr) 3435, 3046, 2927, 1718, 1639, 790 cm^−1^; ^1^H-NMR (CDCl_3_) δ 1.25 (t, *J* = 7.2 Hz, 6H), 2.21 (s, 6H), 3.81 (s, 3H), 4.03 (q, *J* = 7.0 Hz, 4H), 5.22 (s, 1H), 6.87-7.43 (m, 8H), 9.43 (s, 1H); ^13^C-NMR (CDCl_3_) δ 15.25, 19.64, 42.69, 55.73, 62.49, 103.63, 115.84, 117.66, 123.08, 130.41, 134.53, 137.30, 147.59, 156.74, 167.12. Anal. Calcd for C_26_H_29_NO_6_: C, 69.16; H, 6.47, N, 3.10. Found: C, 69.01; H, 6.37; N, 3.00.

*2,6-Dimethyl-4-(4-methylphenyl)-1,4-dihydropyridine-3,5-diethylcarboxylate (**Entry 15, Table 4**)*. Yellow solid; mp 136–137 °C; IR (KBr) 3342, 1693, 1648, 1492, 1210, 770, 612 cm^−1^; ^1^H-NMR (CDCl_3_) δ 1.27 (t, *J* = 7.1 Hz, 6H), 2.29 (s, 3H), 2.32 (s, 6H), 4.13 (q, *J* = 7.1 Hz, 4H), 4.97 (s, 1H), 6.10 (br s, 1H), 7.03 (d, *J* = 7.8 Hz, 2H), 7.23 (d, *J* = 7.8 Hz, 2H); ^13^C-NMR (CDCl_3_) δ 14.29, 19.42, 21.19, 39.14, 59.77, 103.99, 127.59, 128.63, 135.97, 144.33, 145.49, 167.87. Anal. Calcd for C_20_H_25_NO_4_: C, 69.95; H, 7.34, N, 4.08. Found: C, 69.83; H, 7.23; N, 3.98.

*2,6-Dimethyl-4-(hydroxyphenyl)-1,4-dihydropyridine-3,5-diethylcarboxylate (**Entry 16, Table 4**)*. White solid; mp 233–234 °C; IR (KBr) 3330, 1691, 1655, 1490, 1124, 790, 719 cm^−1^; ^1^H-NMR (CDCl_3_) δ 1.24 (t, *J* = 7.2 Hz, 6H), 2.33 (s, 6H), 4.12 (q, *J* = 7.2 Hz, 4H), 5.30 (s, 1H), 5.80 (br s, 1H), 6.66 (d, *J* = 8.9 Hz, 2H), 7.16 (d, *J* = 8.4 Hz, 2H); ^13^C-NMR (CDCl_3_) δ 14.20, 19.84, 39.88, 59.73, 103.81, 127.83, 130.73, 130.38, 142.77, 148.52, 167.36. Anal. Calcd for C_19_H_23_NO_5_: C, 66.07; H, 6.71, N, 4.06. Found: C, 65.93; H, 6.59; N, 3.97.

*2,6-Dimethyl-4-(2-furyl)-1,4-dihydropyridine-3,5-diethanone (**Entry 3, Table 4**)*. Yellowish white solid; mp 176–177 °C; IR (KBr) 3361, 3077, 2985, 1722, 1495, 710 cm^−1^; ^1^H-NMR (CDCl_3_) δ 2.30 (s, 6H), 2.33 (s, 6H), 4.90 (s, 1H), 5.89 (br s, 1H), 6.13–6.21 (m, 2H), 7.50 (m, 1H); ^13^C-NMR (CDCl_3_) δ 19.57, 27.04, 30.27, 107.52, 111.70, 114.21, 143.85, 149.59, 153.50, 197.11. Anal. Calcd for C_15_H_17_NO_3_: C, 69.48; H, 6.61, N, 5.40. Found: C, 69.39; H, 6.52; N, 5.33.

*2,6-Dimethyl-4-(2-furyl)-1,4-dihydropyridine-3,5-dimethylcarboxylate (**Entry 18, Table 4**)*. Yellow solid; mp 149–151 °C; IR (KBr) 3352, 3080, 2992, 1795, 1743, 1460, 1110, 760 cm^−1^; ^1^H-NMR (CDCl_3_) δ 2.28 (s, 6H), 3.76 (s, 6H), 4.98 (s, 1H), 5.90 (br s, 1H), 6.13–6.21 (m, 2H), 7.59 (m, 1H); ^13^C-NMR (CDCl_3_) δ 19.35, 32.56, 55.35, 103.49, 106.08, 110.63, 142.60, 150.99, 153.85, 167.77. Anal. Calcd for C_15_H_17_NO_5_: C, 61.85; H, 5.88, N, 4.81. Found: C, 61.79; H, 5.81; N, 4.77.

*2,6-Dimethyl-4-(2-furyl)-1,4-dihydropyridine-3,5-diethylcarboxylate (**Entry 19, Table 4**)*. Yellow solid; mp 161–162 °C; IR (KBr) 3340, 1695, 1646, 1482, 1365, 1210, 1120, 750, 712 cm^−1^; ^1^H-NMR (CDCl_3_) δ 1.26 (t, *J* = 7.1 Hz, 6H), 2.32 (s, 6H), 4.18 (q, *J* = 7.2 Hz, 4H), 5.21 (s, 1H), 5.92 (br s, 1H), 6.11–6.21 (m, 2H), 7.22 (m, 1H); ^13^C-NMR (CDCl_3_) δ 14.29, 19.44, 33.32, 59.89, 101.38, 104.51, 110.03, 140.78, 145.55, 158.63, 167.61. Anal. Calcd for C_17_H_21_NO_5_: C, 63.94; H, 6.63, N, 4.39. Found: C, 63.82; H, 6.51; N, 4.30.

*2,6-Dimethyl-1-(phenyl)-4-(2-furyl)-1,4-dihydropyridine-3,5-diethylcarboxylate (**Entry 20, Table 4**)*. Brown solid; mp 151–153 °C; IR (KBr) 2985, 1671, 1630, 1065, 872, 740 cm^−1^; ^1^H-NMR (CDCl_3_) δ 1.23 (t, *J* = 7.2 Hz, 6H), 2.21 (s, 6H), 4.26 (q, *J* = 7.0 Hz, 4H), 5.41 (s, 1H), 6.11–6.20 (m, 2H), 7.31–7.69 (m, 6H); ^13^C-NMR (CDCl_3_) δ 15.66, 18.87, 34.18, 62.33, 103.00, 105.42, 111.04, 122.04, 123.11, 128.40, 140.07, 143.77, 151.34, 154.39, 167.62. Anal. Calcd for C_23_H_25_NO_5_: C, 69.87; H, 6.32, N, 3.56. Found: C, 69.82; H, 6.28; N, 3.53.

*2,6-Dimethyl-1-(2′-pyridyl)-4-(2-furyl)-1,4-dihydropyridine-3,5-diethylcarboxylate (**Entry 21, Table 4**)*. White solid; mp 198–199 °C; IR (KBr) 2990, 1693, 1636, 1410, 1035, 970 cm^−1^; ^1^H-NMR (d_6_-DMSO) δ 1.24 (t, *J* = 7.2 Hz, 6H), 2.39 (s, 6H), 4.23 (q, *J* = 7.1 Hz, 4H), 5.37 (s, 1H), 6.14–6.21 (m, 2H), 7.53 (m, 1H), 7.70–8.16 (m, 4H); ^13^C-NMR (d_6_-DMSO) δ 15.22, 19.01, 33.75, 61.87, 104.63, 107.34, 111.02, 118.93, 123.40, 137.66, 143.40, 146.58, 147.52, 149.42, 153.55, 167.09. Anal. Calcd for C_22_H_24_N_2_O_5_: C, 66.65; H, 6.10, N, 7.07. Found: C, 66.59; H, 6.01; N, 7.03.

*2,6-Dimethyl-1-(4-hydroxyphenyl)-4-(2-furyl)-1,4-dihydropyridine-3,5-diethylcarboxy late (**Entry 22, Table 4**)*. Brown solid; mp 68–69 °C; IR (KBr) 3412, 3055, 2970, 1715, 1540, 1472, 1330, 1190, cm^−1^; ^1^H-NMR (CDCl_3_) δ 1.21 (t, *J* = 7.1 Hz, 6H), 1.93 (s, 6H), 4.18 (q, *J* = 7.0 Hz, 4H), 5.26 (s, 1H), 6.11–6.20 (m, 2H), 6.83 (d, *J* = 8.1 Hz, 2H), 6.88 (d, *J* = 8.0 Hz, 2H), 7.52 (m, 1H), 9.82 (s, 1H); ^13^C-NMR (CDCl_3_) δ 15.11, 19.52, 33.29, 61.77, 104.53, 107.22, 112.40, 118.41, 122.00, 132.86, 141.38, 147.26, 152.73, 154.25, 167.58. Anal. Calcd for C_23_H_25_NO_6_: C, 67.14; H, 6.12, N, 3.40. Found: C, 66.99; H, 6.06; N, 3.37.

*2,6-Dimethyl-4-styryl-1,4-dihydropyridine-3,5-dimethylcarboxylate (**Entry 23, Table 4**)*. Yellow solid; mp 119–122 °C; IR (KBr) 3332, 2949, 1701, 1652, 1560, 1492, 1121, 743 cm^−1^; ^1^H-NMR (CDCl_3_) δ 2.35 (s, 6H), 3.71 (s, 6H), 4.54 (d, *J* = 7.2 Hz, 1H), 5.61 (br s, 1H), 6.18 (m, 2H), 7.13–7.39 (m, 5H); ^13^C-NMR (CDCl_3_) δ 14.90, 19.31, 36.17, 51.13, 102.18, 126.34, 126.98, 128.07, 128.28, 131.77, 137.68, 145.43, 167.92. Anal. Calcd for C_19_H_21_NO_4_: C, 69.71; H, 6.47, N, 4.28. Found: C, 69.63; H, 6.42; N, 4.23.

*2,6-Dimethyl-4-cyclohexyl-1,4-dihydropyridine-3,5-diethylcarboxylate (**Entry 24, Table 4**)*. White solid; mp 214 °C; IR (KBr) 2996, 1770, 1465, 1378, 1240 cm^−1^; ^1^H-NMR (CDCl_3_) δ 1.24 (t, *J* = 6.9 Hz, 6H), 1.32–1.77 (m, 11H), 2.24 (s, 6H), 3.85 (d, *J* = 5.8 Hz, 1H), 4.21 (q, *J* = 8.1 Hz, 4H), 5.81 (br s, 1H); ^13^C-NMR (CDCl_3_) δ 15.08, 19.23, 26.21, 26.64, 32.69, 38.36, 45.85, 50.97, 102.02, 144.78, 168.74. Anal. Calcd for C_19_H_29_NO_4_: C, 68.03; H, 8.71, N, 4.18. Found: C, 68.05; H, 8.66; N, 4.12.

*2,6-Dimethyl-4-isopropyl-1,4-dihydropyridine-3,5-dimethylcarboxylate (**Entry 25, Table 4**)*. Yellow solid; mp 164–166 °C; IR (KBr) 3334, 1705, 1652, 1570, 1462, 1375, 1220, 910 cm^−1^; ^1^H-NMR (CDCl_3_) δ 0.75 (d, *J* = 6.9 Hz, 6H), 1.64 (sep, *J* = 6.8 Hz, 1H), 2.31 (s, 6H), 3.72 (s, 6H), 3.92 (d, *J* = 5.5 Hz, 1H), 5.65 (s, 1H); ^13^C-NMR (CDCl_3_) δ 19.31, 19.96, 35.86, 39.04, 51.09, 102.53, 145.22, 169.10. Anal. Calcd for C_14_H_17_NO_4_: C, 62.90; H, 7.92, N, 5.24. Found: C, 62.78; H, 7.84; N, 5.17.

## 4. Conclusions

Application of microwave technology to rapid synthesis of biologically significant heterocyclic molecules under solvent-free conditions is very promising and challenging. The ultimate aim, of course, is to use no solvent at all and to conduct the reactions under solvent-free conditions [74]. Development of cleaner technologies is a major emphasis in green chemistry. The combination of solvent-free reaction conditions and microwave-irradiation is used as an eco-friendly approach for the synthesis of a variety of products and this generally leads to large reductions in reaction times, enhancements of conversions, and changes of selectivity. 

There is growing interest in the one-pot three component synthesis of 1,4-dihydropyridines because of the significant importance of this scaffold in preparing a wide variety of biologically and pharmacologically active molecules. On this basis we have developed an extremely rapid, convenient and environmentally benign route for the one-step synthesis of 1,4-dihydropyridines. The present methodology offers attractive features such as shorter reaction times, milder conditions, and simplicity of the reaction as well as excellent yield of the products. This reaction will be applicable to the synthesis of various organic compounds of medicinal interest. 
